# Impact of In Utero Exposure to Antiepileptic Drugs on Neonatal Brain Function

**DOI:** 10.1093/cercor/bhab338

**Published:** 2021-09-29

**Authors:** Anton Tokariev, Michael Breakspear, Mari Videman, Susanna Stjerna, Lianne H Scholtens, Martijn P van den Heuvel, Luca Cocchi, Sampsa Vanhatalo

**Affiliations:** Baby Brain Activity Center (BABA), Department of Clinical Neurophysiology, New Children's Hospital, HUS Imaging, Helsinki University Hospital and University of Helsinki, Helsinki, Finland; Neuroscience Center, Helsinki Institute of Life Science, University of Helsinki, Helsinki, Finland; School of Psychology, College of Engineering, Science and the Environment, University of Newcastle, Callaghan, New South Wales, Australia; School of Medicine and Public Health, College of Health and Medicine, University of Newcastle, Callaghan, New South Wales, Australia; Baby Brain Activity Center (BABA), Department of Clinical Neurophysiology, New Children's Hospital, HUS Imaging, Helsinki University Hospital and University of Helsinki, Helsinki, Finland; Department of Pediatric Neurology, New Children's Hospital, Helsinki University Hospital and University of Helsinki, Helsinki, Finland; Baby Brain Activity Center (BABA), Department of Clinical Neurophysiology, New Children's Hospital, HUS Imaging, Helsinki University Hospital and University of Helsinki, Helsinki, Finland; Neuroscience Center, Helsinki Institute of Life Science, University of Helsinki, Helsinki, Finland; Department of Complex Trait Genetics, Center for Neurogenomics and Cognitive Research, Faculty of Science, Vrije Universiteit Amsterdam, Amsterdam Neuroscience, Amsterdam, the Netherlands; Department of Complex Trait Genetics, Center for Neurogenomics and Cognitive Research, Faculty of Science, Vrije Universiteit Amsterdam, Amsterdam Neuroscience, Amsterdam, the Netherlands; Department of Child Psychiatry, Amsterdam University Medical Center, Amsterdam Neuroscience, Amsterdam, the Netherlands; Department of Genetics and Computational Biology, QIMR Berghofer Medical Research Institute, Brisbane, Australia; Baby Brain Activity Center (BABA), Department of Clinical Neurophysiology, New Children's Hospital, HUS Imaging, Helsinki University Hospital and University of Helsinki, Helsinki, Finland; Neuroscience Center, Helsinki Institute of Life Science, University of Helsinki, Helsinki, Finland

**Keywords:** antiepileptic drug, brain network, infant, neurodevelopment, neurology

## Abstract

In utero brain development underpins brain health across the lifespan but is vulnerable to physiological and pharmacological perturbation. Here, we show that antiepileptic medication during pregnancy impacts on cortical activity during neonatal sleep, a potent indicator of newborn brain health. These effects are evident in frequency-specific functional brain networks and carry prognostic information for later neurodevelopment. Notably, such effects differ between different antiepileptic drugs that suggest neurodevelopmental adversity from exposure to antiepileptic drugs and not maternal epilepsy per se. This work provides translatable bedside metrics of brain health that are sensitive to the effects of antiepileptic drugs on postnatal neurodevelopment and carry direct prognostic value.

## Introduction

The growth of brain networks in utero sets the foundation for lifelong neurocognitive performance (Kostović et al. 2019; Thomason 2020). Fetal brain development is sensitive to a wide range of adversities in the in utero environment, including maternal illness and its pharmacological treatment (Thomason 2020). This is particularly true of epilepsy which is common among women of child-bearing age (Meador et al. 2013). Therefore, understanding mechanisms and impact of early adversities on the emergence of functional brain networks is a key priority in neonatal medicine. Developing bedside tests of postnatal brain health is, likewise, a priority to enable early detection and targeted intervention.

The development of fetal brain networks is critically dependent on endogenous neural activity (Kirischuk et al. 2017; Molnár et al. 2020), which supports neuronal survival, shapes the formation of macroscopic networks (Hanganu-Opatz et al. 2021) and influences the functional dynamics they support (Benders et al. 2015; Luhmann et al. 2016). These patterns of neural activity can be affected by external factors and physiological challenges (Kirischuk et al. 2017; Mizuno et al. 2021), including the in utero exposure to drugs (Videman et al. 2016b; Videman et al. 2017). Specifically, antiepileptic drugs (AED) administered maternally carry an established risk of disrupting early neurodevelopment (Meador et al. 2009; Kellogg and Meador 2017; Veroniki et al. 2017). The therapeutic actions of these drugs inhibit brain networks via modulation of ion channel activity or synaptic transmitter release (Macdonald and Kelly 1995; Mantegazza et al. 2010). Ion channel functions are essential for the long-range synchronization between neural populations (Destexhe and Sejnowski 2003; Rama et al. 2015; Dewell and Gabbiani 2019), hence fetal exposure to AED may jeopardize fetal brain activity and consequently neurodevelopment.

Although preclinical studies identify channelopathies (Smith and Walsh 2020) and other mechanisms (Kirischuk et al. 2017; Bikbaev et al. 2020), the exact pathophysiological effects of AEDs in humans are not understood (Smith and Walsh 2020). Recent studies in humans have suggested that channelopathies during gestation are different from those occurring postnatally (Smith and Walsh 2020). The analysis of functional network activity in neonates after in utero AED exposure offers a unique opportunity window into brain health and thus a route to explore such mechanisms. In particular, cortical activity dynamics during sleep is a sensitive indicator of early neurodevelopmental perturbations such as those arising from in utero AED exposure (Videman et al. 2016b; Tokariev et al. 2019a; Tokariev et al. 2019b). Infants spend most of their time asleep, switching between active sleep (AS) and quiet sleep (QS), and sleep-related cortical activity shapes brain development and influences developmental outcomes (Dooley et al. 2019; Tokariev et al. 2019a; Blumberg et al. 2020a; Molnár et al. 2020; Hanganu-Opatz et al. 2021).

Prior findings on large-scale amplitude correlations have demonstrated robust whole-cortex network reconfigurations between sleep states in healthy infants (Tokariev et al. 2019a). Here, we extend our previous work by assessing sleep-related dynamics in phase–phase coupling (PPC) networks, which reflects neuronal communication at higher temporal precision compared with amplitude correlations (Engel et al. 2013; Tokariev et al. 2019b; Siems and Siegel 2020). Due to the higher temporal resolution and presumably tighter relationship to neuronal firing patterns, PPC may be more sensitive than amplitude–amplitude correlations (AAC) in assessing effects of environmental adversities such as drug exposures on early neuronal development.

We hypothesized that AEDs would alter the neurodevelopment of in utero cortical networks in manner that was frequency-specific and unique to AED class. To test this hypothesis, we evaluated the putative effect of in utero AED exposure on large-scale, sleep-related cortical dynamics across a range of different carrier frequencies. This was achieved by applying advanced methods for extracting functional brain networks from sleep-related scalp electroencephalography (EEG) across broad frequency ranges (Tokariev et al. 2019a; Tokariev et al. 2019b). We also evaluated if changes in AED-driven cortical network dynamics were related to their underlying histology and pre-empted later neurocognitive outcomes.

## Materials and Methods

### General Overview of the Study Design

**Figure 1:**
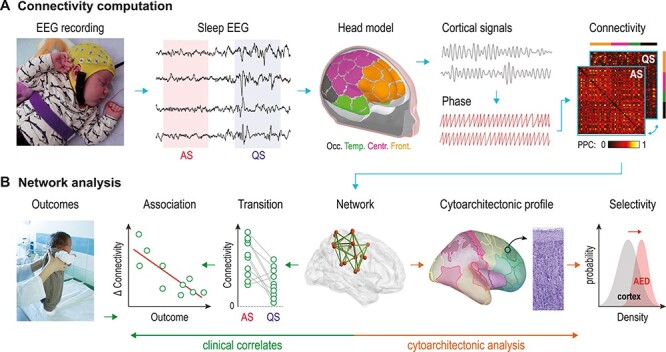
Analytical pipeline. (A) Scalp EEG was recorded during sleep at term-equivalent age from 2 groups of infants: infants exposed to in utero treatment with antiepileptic drugs (AED, *N* = 46), and HC (*N* = 61). Epochs of active sleep (AS) and quiet sleep (QS) were source transformed into 58 cortical parcels using a realistic infant head model. Functional connectivity was estimated using pairwise phase–phase correlation (PPC) between all cortical parcels. (**B**) Network-based statistics were applied to cortical functional connectivity matrices to isolate networks showing main effects of sleep phase and group, as well as sleep-by-group interactions. The difference in connectivity between sleep states yielded a measure of network dynamics (∆ connectivity = AS − QS). This measure was used to study associations with clinical outcomes. Cytoarchitectonic profiles of AED-induced networks were contrasted to the whole brain to assess whether drug effects overlap with cortical histological properties. Nissl stain image of infant cortex is modified from the (Judaš et al. 2011). Source of the photograph in A is BABA Center; source of the photograph in B is University of Helsinki, Linda Tammisto.

We compared scalp EEG recordings in sleeping newborn infants exposed to AED in utero to infants with no medical history and without drug exposure (healthy controls, HC). After EEG data reconstruction into cortical space signals, frequency-specific cortical networks were computed by estimating PPC. Statistical methods adapted for networks were then employed to identify the main effects of sleep states and group assignment, their interactions and their associations with later neurocognitive performance. Finally, we assessed the putative link between networks affected by in utero AED exposures and the infant’s brain cytoarchitecture (Fig. 1).

### Subjects and Background Information

Infants in both groups were born full-term: AED at 40.1 ± 1.4 weeks and HC at 40.3 ± 1.1 weeks (mean ± standard deviation; SD) with no significant difference in gestational age (*P* = 0.68, Wilcoxon rank-sum test). AED cohort included infants exposed to monotherapy (one AED type; *N* = 35) and polytherapy (POLY, exposed to more than one AED; *N* = 11). In turn, monotherapy subgroup included infants exposed to different drug types: carbamazepine (CBZ, N = 9), oxcarbazepine (OXC, *N* = 8), levetiracetam (LEV, *N* = 7), lamotrigine (LTG, *N* = 6), valproic acid (VPA, *N* = 4), and topiramate (TPM, *N* = 1).

Data on recruitment, drug exposure, epilepsy diagnoses, and seizure status of the mothers were obtained prospectively during outpatient visits (every trimester and postnatally). There were no significant differences between AED and HC in the duration of the pregnancy, the history of maternal folic acid supplementation, cigarette smoking, alcohol consumption, age, and parity.

The ethics committee of the Helsinki University Hospital (Finland) approved the study. Written informed consent was obtained from all the mothers. Background information, results of neurological examinations of the infants, and neurocognitive evaluation of the mothers have been described in detail in a previous study (Videman et al. 2016b).

### E‌EG Recordings

Scalp EEG was measured at conceptional age of 42.1 ± 0.9 weeks in AED and 42.2 ± 0.9 weeks in HC groups (or 2.0 ± 1.0 and 2.0 ± 1.1 weeks from birth, respectively; mean ± SD for all) with no significant difference (*P* = 0.51, Wilcoxon rank-sum test). The EEG recording was performed during daytime sleep with a NicOne EEG amplifier (Cardinal Healthcare/Natus, USA), using initial sampling frequency (Fs) of 250 Hz or 500 Hz. The neonatal Waveguard caps had 20–32 sintered Ag/AgCl electrodes (ANT-Neuro, Germany) placed according to the 10–20 international system. For further analysis, the same 19 channels were selected from all subjects: Fp1, Fp2, F7, F3, Fz, F4, F8, T7, C3, Cz, C4, T8, P7, P3, Pz, P4, P8, O1, and O2.

EEG epochs for the network analyses were selected from both neonatal sleep (vigilance) states, AS and QS. They were identified visually using the standard combination of electrophysiological and behavioral measures (André et al. 2010), including polygraphic channels (submental electromyogram, electrocardiogram, electrooculogram, and respiration sensor). EEG signal during AS is known to exhibit continuous fluctuations, while polygraphic channels show irregular respiration and occasional eye movements. Conversely, EEG signal during QS is characteristically discontinuous, while respiration is markedly regular.

### E‌EG Preprocessing

For the quantitative analysis, 3 min of artifact-free EEG (see also Supplementary Material) for both AS and QS in each infant were selected. These epochs were accumulated by considering 6 equidistant 30-second-long windows across the whole recording. This signal length was chosen as a compromise between maximal subject inclusion versus reliable estimation of network connectivity. Subjects lacking sufficient epoch lengths or with technically low data quality were excluded. This led to final sample sizes *N* = 46 subjects in AED and *N* = 61 subjects in HC. The original cohorts included *N* = 52 (AED) and *N* = 68 (HC) subjects.

Selected epochs were prefiltered within frequency band 0.4–45 Hz, down-sampled to *F*s = 100 Hz, and converted to average montage. Next, based on our previous works (Videman et al. 2016b; Tokariev et al. 2019a), these data were filtered into 4 frequency bands of interest: 0.4–1.5 Hz (low delta), 1.5–4 Hz (high delta), 4–8 Hz (theta), and 8–13 Hz (alpha). All bandpass filtering was implemented by means of low-pass and high-pass filter pairs with corresponding cut-off frequencies. Butterworth type filters with stop-band attenuation of 20 dB were designed using Matlab function *designfilt*. Filtering of EEG epochs was done in forward–backward directions to compensate for phase delays caused by infinite impulse response filters.

### Reconstruction of Cortical Signals

Preprocessed EEG sleep epochs were further used to compute cortical signals. To do so, a three-shell infant head model was employed wherein scalp, skull and intracranial volume boundaries (2562 vertices per surface) were generated from manually segmented magnetic resonance images (MRI) of an infant at term age. Based on previous studies (Despotovic et al. 2013; Odabaee et al. 2014; Tokariev et al. 2016a), conductivities of these compartments were set to 0.43 S/m, 0.2 S/m, and 1.79 S/m respectively. Source space had 8014 orthogonal to surface dipoles located on rescaled and smoothed (to match anatomically plausible brain geometry at this age) cortical template. In the model, EEG sensors were placed according to the experimental setup. First, scalp EEG was converted into source signals using the symmetric boundary element method (Gramfort et al. 2010) for forward modeling and dynamic statistical parametric mapping (Dale et al. 2000) for inverse modeling in Brainstorm software (Tadel et al. 2011). Further, all sources were collapsed into 58 cortical regions according to the parcellation scheme (Fig. 1A) developed to analyze functional brain connectivity in infants (Tokariev et al. 2019b). All the parcels were labeled into 4 groups depending on their location: frontal, central, temporal, and occipital. Such division mainly corresponds to their overlap with the brain anatomical lobes with the only difference that central parcels comprised parietal areas and few frontal areas that are proximal to the central sulcus. Finally, neural activity of each area was computed as an average of underlying source signals weighted according to their ability to represent cortical activity in the model (Tokariev et al. 2019b).

In order to optimize spatial accuracy in source reconstruction, the electrode placements were standardized by using a combination of EEG cap and its placing according to well established anatomical landmarks (ears, nasion, inion). Recent work using infant MRI-based simulations has shown, however, that using anatomical landmarks may result in some localization error (up to 20 mm) with respect to cortical space (Kabdebon et al. 2014). To mitigate this problem, we used fairly large cortical source aggregates (average parcel diameter 25 mm). Analyses were performed at both parcel level and an even coarser regional level. In addition, our work presents findings in widespread networks rather than in individual parcels. We assume therefore that the findings are not significantly confounded by the limits of accuracy in electrode positioning.

### The Computing of Functional Connectivity

Functional connectivity (interactions) between pairs of cortical regions was computed by assessing phase synchrony in the corresponding cortical signals (Fig. 1A). For this purpose, a debiased weighted phase lag index (wPLI; Vinck et al. 2011) was used as this measure is relatively robust to volume conduction effects (Palva et al. 2018). Connectivity matrices (comprise all possible pairwise interactions) were computed for each subject, sleep state, and frequency band. These empirical matrices were corrected based on results of computer simulations aimed to exclude “noisy” connections (Tokariev et al. 2019b). In brief, for each possible connection, 500 iterations generating perfectly synchronous signals for corresponding pairs of underlying parcels (so that wPLI = 1) and unsynchronised signals for all the rest were ran. Next, sensor-space data were computed using a forward solution and then cortical signals were reconstructed back. Mean wPLI for the “connection of interest” across 500 iterations was taken as a measure of its fidelity and all other values were accumulated into a pool of surrogates representing “noise”. Finally, each edge-specific fidelity value was tested against the 99th percentile of surrogates from all iterations. This led to a binary mask (with 1 for wPLI above the threshold and 0 for the opposite case) that was applied for empirical connectivity matrices. This procedure excluded from further analysis the same 525 edges from each matrix that cannot be reliably estimated with a particular 19-channel sensor set.

### Cortical Network Analysis

Cortical regions were considered as nodes and strength of phase synchronization (wPLI values) between regions as edges of functional networks (Fig. 1B). Network-based statistics (NBS; Zalesky et al. 2010) was used to isolate cortical networks showing main effects of sleep (AS vs. QS) and group (AED vs. HC), as well as sleep-by-group interaction. For the AED subgroups, only sleep-by-group interactions were computed. Analyses were performed for each frequency band of interest. A *t*-threshold was set to 2.5 and applied to each edge of interest (above). A permutation-based correction for family-wise error (FWE) rate (5000 permutations, alpha probability <0.05) was ascribed to cortical networks with edge values above the initial threshold. The effect size for all interaction networks was estimated using Cohen’s *d* (average *t*-statistics divided by the square root of the degrees of freedom).

### Clinical Neurocognitive Assessments

#### Newborn Neurological Performance

Infants were assessed at term-equivalent age of 42.1 ± 0.8 weeks using the Hammersmith Neonatal Neurological Examination (HNNE; Dubowitz et al. 1999). Initially, HNNE includes series of semiquantitative tests that characterize the following neurological domains: reflexes, movements, posture tonus, tone patterns, abnormal signs, orientation and behavior. The dimensionality of HNNE scores was reduced by applying principal component analysis (PCA) as described before (Tokariev et al. 2019b). The first two components (hereafter called C1 and C2) combined visual alertness, head raising in prone, and extensor tone. Comparison to later neurodevelopmental outcomes has shown earlier that C1 associates with later motor development, whereas C2 is linked to later cognitive and social development (Tokariev et al. 2019b).

#### Neurocognitive Development at 2 years

Cognitive development was evaluated at the age of 24.3 ± 0.3 months by a psychologist using neurodevelopmental assessment according to Bayley Scales of Infant and Toddler Development (BSID-III; Bayley 2006). The measures used in our study are compound scores from a larger set of individual test items, representing four major domains: cognitive, receptive communication, expressive communication, and fine motor were employed. These scores are the finest resolution that is commonly approved in international practices when assessing their relationships to other measures, such as the EEG-derived networks in our study (Del Rosario et al. 2021).

Both neurological and neurocognitive assessments in two groups were done at the same research laboratory (BABA Center, Helsinki).

### Analysis of Network-to-Outcome Relation

Networks showing a significant sleep-by-group interaction were tested for their relationship to clinical outcomes in the infants exposed to AED (Fig. 1B). Individual sleep-specific (AS and QS) connectivity matrices were masked with a binary template resulting from the NBS analysis. Next, the group differences in mean connectivity strengths between sleep states were computed as ∆ = AS − QS. These delta values were correlated (two-tailed Spearman’s test) to two sets of clinical scores. First, neurological performance at the time of EEG (group SD was ±2 days from the recordings) was assessed by using the C1 and C2 scores (data available for *N* = 46 infants). Second, the relationship with neurocognitive outcomes measured at 2 years of age was tested. These outcomes reflect important aspects of neurocognitive performance (cognitive, receptive language, expressive language, and fine motor scores; data available for *N* = 44 subjects). Resulting *P*-values for each frequency-specific AED network (Fig. 3A) were grouped according to the assessment type (2 tests vs. neurological scores, and 4 tests vs. neurocognitive scores) and corrected using Benjamini–Hochberg false discovery rate (FDR) procedure.

### Cytoarchitectonic Infant Brain Atlas

The infant’s cytoarchitectonic brain atlas was constructed using a digital version of original Von Economo-Koskinas atlas (Scholtens et al. 2018), further subdividing regions FA, PB, and PC into receptive fields for specific body parts (leg, trunk, hand, and head). Corresponding cytoarchitectonic features of infant cortical regions at the age of one month (most proximal time point to the analysis groups) were collected from the works of Conel (Conel 1941; Shankle et al. 2000). For our analyses (Fig. 5), layer-specific neural densities were used as this feature was described for all cortical regions at this age, allowing for a whole-cortex mapping.

### Linking Alerted Connectivity to Cytoarchitectonic Features

The cytoarchitectonic atlas was projected onto the source space used for the connectivity analysis. Each source was associated with six values, corresponding to the estimated neuronal densities of six cortical layers. These data were used to compute density distributions across the whole cortex within each layer (Fig. 1B). Next, cytoarchitectonic cortical profiles were derived for networks that showed a significant sleep-by-group interaction (monotherapy AED subgroups vs. HC, Fig. 4A). The network’s neural density distributions (within each layer) were compared with the whole-cortex distributions using a two-sample Kolmogorov–Smirnov test (Matlab function kstest2, with alpha level set to 0.05). The resultant Kolmogorov–Smirnov statistic (*D,* which shows the maximal distance between the cumulative distribution functions) was further tested against *N* = 1000 surrogate *D-*values computed between the whole cortex and the cytoarchitectonic profiles of randomly selected networks of the same size as AED-induced one. Probability values for each test were ascribed based on probability distributions of the surrogate *D*-values. Finally, all *P*-values (5 monotherapy networks × 6 layers = 30 tests) were corrected with Benjamini–Hochberg FDR procedure.

### Analysis Software

Clinical EEG review and epoch selection was performed using the software NicoletOne EEG reader (Cardinal Healthcare, Natus, USA). Cortical sources from EEG data were computed using Brainstorm software (https://neuroimage.usc.edu/brainstorm). Custom Matlab scripts implementing source-level connectivity is available at https://github.com/babyEEG/AED-exposed-infants. For network analysis, NBS toolbox (https://www.nitrc.org/projects/nbs) was employed. For statistical analysis standard Matlab functions (version R2016b) and JASP software (https://jasp-stats.org) were used. Network visualizations were done in BrainNet Viewer (Xia et al. 2013) (https://www.nitrc.org/projects/bnv).

### Data Availability

With this work, we provide Matlab code for source-level connectivity analysis and frequency-specific connectivity matrices used in this study. Original clinical EEG data and neurocognitive performance assessments can be available after making data sharing agreement with Helsinki University Hospital.

## Results

### Effect of Sleep States on Cortical Dynamics

**Figure 2:**
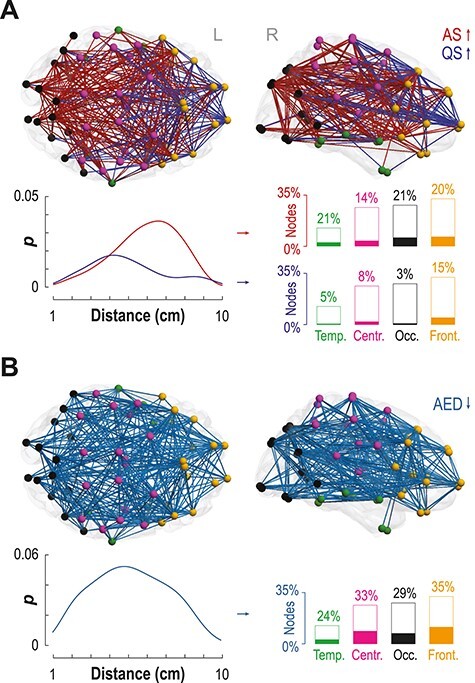
Effects of sleep state and AED exposure on cortical networks at high-delta frequency (1.5–4 Hz). (**A**) The effect of sleep (AS vs. QS) highlights 2 spatially distinct cortical networks (*P*_FWE_ < 0.0001). A dominant occipital-central network (red) supports active sleep (AS) whereas a frontocentral network (blue) is expressed in quiet sleep (QS). Probability (*P*) distribution plots of Euclidean distances between parcel centroids indicate that AS networks are dominated by long-range projections. In contrast, QS networks involve a larger number of short-range connections. Bar plots show the portion of these connections in each cortical area relative to the whole network (height) and their filling reflects involvement in the given sleep-related networks. (**B**) The main effect of group (AED vs. control) revealed a widespread reduction in connectivity strength in the AED infants (*P*_FWE_ < 0.01). The detected network shows no clear preference for connection lengths (distribution of connection distances) or cortical areas. Cortical regions are marked with different colors: occipital (black), temporal (green), central (purple), and frontal (orange).

The main effect of sleep (AS vs. QS) revealed robust functional network differences. There was a marked reorganization of cortical networks along the posterior–anterior cortical axis, with frequency-specific variations in network topology (Fig. 2A). The most salient sleep stage difference occurred in the high delta (1.5–4 Hz) frequency range: AS was linked to stronger connectivity in long-range connections (red, Fig. 2A) whereas QS was associated with higher connectivity in short-range frontocentral connections (blue, Fig. 2A; *P_FWE_* < 0.0001 for both; paired two-tailed *t*-tests). The QS networks at neighboring low delta (0.4–1.5 Hz) and theta (4–8 Hz) frequencies had comparable topology (blue in Supplementary Fig. S1, both *P_FWE_* < 0.0001), suggesting a broadband effect of QS on cortical connectivity (Tokariev et al. 2019a). In contrast, the AS network in the theta band (red in Supplementary Fig. S1, *P_FWE_* < 0.0001) involved higher connectivity between central and occipital cortices than QS. Sleep-induced connectivity changes in the alpha band (8–13 Hz) involved relatively few connections (Supplementary Fig. S1, *P_FWE_* < 0.048 for both AS > QS and AS < QS).

### Exposure to In Utero AED Reduced Connectivity in Networks Supporting Sleep

Comparing cortical networks activity between infant groups (AED vs. HC) showed a significant reduction in functional connectivity strength in the AED infants across both sleep states. This comprised a diffuse network in the high-delta frequency range (Fig. 2B, *P_FWE_* = 0.01, unpaired two-tailed *t*-tests). The attenuated connectivity encompassed almost one-third of all possible cortical connections. Two occipital connections in the alpha frequency range showed increased connectivity in the AED group (Supplementary Fig. S2; *P_FWE_* = 0.01, unpaired two-tailed *t*-tests). Other frequency bands did not show any significant differences between the two groups.

### AED Exposure Affects the Dynamics of Cortical Sleep Networks

**Figure 3:**
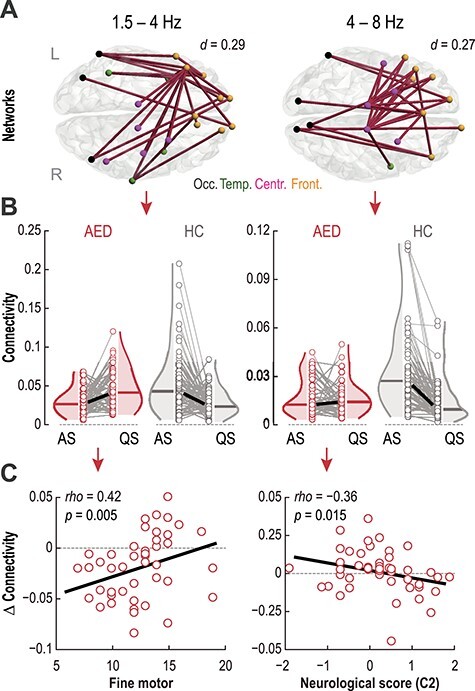
General effects of AED exposure on cortical sleep networks and behavioral outcomes. (**A**) Networks with predominantly frontal projections showed significant sleep-by-group interactions at high delta (1.5–4 Hz) and theta (4–8 Hz) frequencies (*P*_FWE_ = 0.013 with Cohen’s *d* = 0.29 and *P*_FWE_ = 0.008 with Cohen’s *d* = 0.27, respectively). The centroids of cortical parcels are coded with different colors according to their location: occipital (black), temporal (green), central (purple), and frontal (orange). (**B**) In these 2 networks, AED and HC groups showed opposite mean connectivity strength in AS and QS. The thin gray lines show individual changes in connectivity strength whereas the thick lines show the group medians. (**C**) Spearman’s correlations between changes in connectivity strength as a function of sleep states (∆ = AS − QS) within the 2 “interaction” networks (panel A) and behavioral outcomes at the level of the whole AED group. Individual changes in sleep-induced connectivity in the high-delta frequency correlates with fine motor performance at 2 years. Changes in the theta frequency network negatively correlate with the compound neurological performance score C2 at term age.

A sleep-by-group interaction was observed in two cortical networks, one in the high delta (1.5–4 Hz) and the other in the theta (4–8 Hz) frequency range (Fig. 3A; *P_FWE_* = 0.013 and *P_FWE_* = 0.008, respectively). The networks in each of these frequency bands possess distinct topological differences. Changes in mid- and long-range connections dominated the network effects in the high-delta frequency (left on Fig. 3A). Conversely, in the theta frequency, the network effects occurred in short-range connections linking frontal to central cortices and long-range connections linking these brain regions to occipital cortices (right on Fig. 3A). Crucially, AED exposure “reversed” the direction of the changes in network strengths between sleep states (Fig. 3B). For example, in the AED group, high-delta and -theta networks increased from AS to QS, whereas the opposite change occurred in the control infants (Fig. 3B).

**Figure 4:**
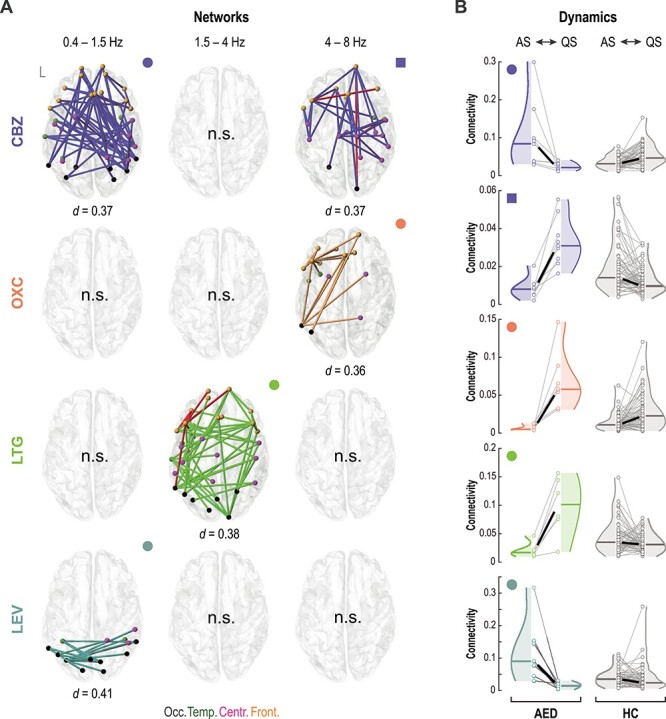
Drug-specific effects on sleep-related cortical networks. (A) Cortical networks showing a sleep-by-subgroup interaction. The centroids of each cortical parcel are color-coded as a function of their overlap in the cortical region: occipital (black), temporal (green), central (purple), and frontal (orange). AED subgroups are color-coded as follows: carbamazepine (CBZ, blue; *N* = 9), oxcarbazepine (OXC, light orange; *N* = 8), lamotrigine (LTG, light green; *N* = 6), levetiracetam (LEV, aquamarine; *N* = 7). Connections that overlap with the common effect (Fig. 3A) are shown with red color. The effect size for each network was estimated using Cohen’s d. Note that “n.s.” denotes no significant sleep state-by-group interaction. (**B**) Composite connectivity changes in each cortical network. The violin plots are coded with symbols indicating the given network topology in panel A. Thin gray lines connect individual means of network strength, and thick lines show group medians.

We also asked if these “general” AED-related sleep-by-group interactions might be driven by specific medications. To test this hypothesis, we compared changes in connectivity strength within the two networks of interest between five major AED subgroups: carbamazepine (CBZ), oxcarbazepine (OXC), levetiracetam (LEV), lamotrigine (LTG), and polytherapy (POLY). Although acknowledging the relatively small sample in each subgroup, these analyses did not support the hypothesis that the “general” effects of AED are driven by a specific drug type (*P* = 0.3 for the high delta and *P* = 0.7 for the theta network; Supplementary Fig. S3).

### AED-Induced Changes in Cortical Functional Connectivity Relate to Behavioral Outcomes

We next assessed the behavioral relevance of the effects of AED on sleep-related networks. Specifically, we tested for putative associations between connectivity changes and neurological performance (2 measures) near the time of EEG recording, as well as the neurocognitive outcomes (4 measures) assessed at 2 years of age (Methods). We found a significant correlation between changes within high-delta network and fine motor behavior at 2 years (Fig. 3C; Spearman’s *rho* = 0.42, *P* = 0.005, *P_FDR_* = 0.02). Moreover, the C2 neurological performance score, at the time of EEG recording, was negatively correlated to the theta network dynamics (Fig. 3C; Spearman’s *rho* = −0.36, *P* = 0.015; *P_FDR_* = 0.03). Other scores did not show significant correlations to these networks (Supplementary Table S1), suggesting a selective effect of AED on cortico-behavioral associations.

### Drug-Specific Effects Show Different Spatial Patterning that Links to Future Neurodevelopment

AEDs target different molecular mechanisms (Smith and Walsh 2020) and may thus cause distinct developmental channelopathies, leading to spatially distinct changes in sleep-related networks. We next tested this hypothesis by assessing if the topology of sleep-state networks varies between AEDs.

AED-specific effects were confined to lower (≤8 Hz) frequencies (Fig. 4A), with distinct sleep-state changes across main monotherapy subgroups (Fig. 4B). In the CBZ subgroup, two cortical networks showed opposite changes in connectivity as a function of sleep states compared with HC (Fig. 4A, blue): a topologically widespread network in the low-delta frequency band (*P_FWE_* = 0.025) and a predominantly right lateral network in the theta range (*P_FWE_* = 0.002). In the OXC subgroup, a sleep-by-group interaction was comprised by a mainly left-sided network in the theta range (Fig. 4A, orange; *P_FWE_* = 0.016). The LTG subgroup showed a widespread network effect in the high-delta range (Fig. 4A, green; *P_FWE_* = 0.003). In the LEV subgroup, a sleep-by-group interaction was confined to the occipital cortex (Fig. 4A, aquamarine; *P_FWE_* = 0.033, low-delta frequency range). The small group size of the VPA infants (*N* = 4) precluded a meaningful assessment of the sleep-by-group interaction. There was no significant effect (*P_FWE_* = 0.07) in the infant subgroup exposed to multiple AEDs (POLY). Notably, only very few edges (red in Fig. 4A) were shared by more than one AED types. Taken together, these findings indicate that exposures to specific AEDs lead to distinct spatiotemporal network effects.

To explore clinical significance of these effects, we computed correlations between sleep-induced connectivity changes and clinical neurodevelopmental scores. Consistent with previously known neurodevelopmental effects (Kellogg and Meador 2017; Veroniki et al. 2017), the AED-affected functional cortical networks were linked to newborn neurological performance and later language-related neurocognitive performance (Supplementary Fig. S4).

### The Topology of AED-Affected Networks Map onto Infants Cortical Cytoarchitectonic Profiles

**Figure 5:**
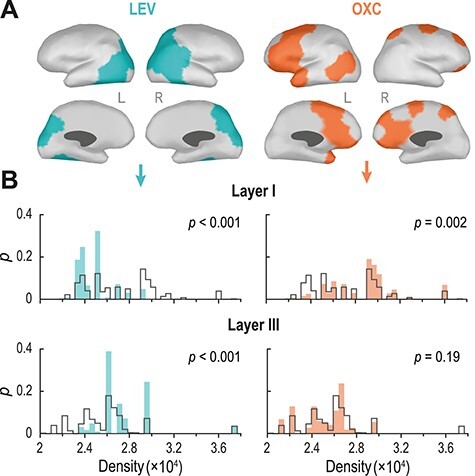
AED effects on functional networks link to cortical cytoarchitectonic profiles. (A) Cortical involvement of networks that show sleep-by-subgroup interactions (Fig. 4A) for levetiracetam (LEV; aquamarine) and oxcarbazepine (OXC; orange). (B) Normalized probability distributions (*P*) of neural densities (neurons per 1 mm^3^) within these areas for layer I (top) and layer III (bottom) are shown with the same colors. Distributions of neural densities across the whole cortex in the corresponding layers are shown with gray lines. In layer I, the neuronal densities were relatively lower in the LEV-related network and higher in the OXC-related networks. In layer III, LEV-affected cortices showed higher neuronal densities, whereas OXC-related networks were no different from the other cortex. The density distributions were compared with two-sample Kolmogorov–Smirnov tests followed by a permutation test (*N* = 1000).

We further assessed possible cellular correlates for the observed drug-specific effects on functional cortical networks. We analyzed the spatial distribution of “drug-specific” networks effects (Fig. 4A) with respect to the estimated neuronal density in each cortical layer, obtained from the infant Conel cytoarchitectonic atlas (Conel 1941). The distributions of neuronal densities within cortical regions composing each functional network were compared with the average neuronal densities across all cortical regions. Cortical regions in the LEV-affected network (aquamarine on Fig. 5A) had a relatively lower neuronal density in *layer I* and a higher density in *layer III* (left on the Fig. 5B; *P_FDR_* = 0.015, for both). In contrast, regions in the OXC network (orange on Fig. 5A) showed a high neural density in *layer I* (right on the Fig. 5B; *P_FDR_* = 0.02).

## Discussion

Here we show that in utero exposure to pharmacological treatment with AEDs influences the functional organization of large-scale cortical networks in the neonate. These neurophysiological effects impact brain networks activity during sleep, are specific to the type of AED, reflect cortical cytoarchitectonic profiles, and carry prognostic information. Our findings provide the first detailed link between in utero AED exposure, topographic changes in cortical sleep networks and childhood neurocognitive performance (Kirischuk et al. 2017; Chini et al. 2020). In doing so, this work advances our fundamental understanding of the mechanisms of adverse AED exposure in utero. In addition, the cortical networks—derived from bedside EEG—provide novel biomarkers for further clinical and translational discovery.

Our present results complement our prior work comparing global connectivity levels of PPC sleep networks (Tokariev et al. 2016b; Tokariev et al. 2019b). Here, we extend previous findings by characterizing the detailed network topology at the level of cortical sources. This discloses spatially delimited group differences in the frequency-specific networks that could not be seen in prior global-level analysis of scalp EEG signals. The present PPC network effects appear to be at lower frequencies than what detected for source-level AAC networks (Tokariev et al. 2019a). The PPC type connectivity requires far higher precision in neuronal communication synchrony compared with AAC (Engel et al. 2013; Tokariev et al. 2016b), which is robust already at a very early developmental stage (Koolen et al. 2016). Hence, the structural networks supporting higher frequency PPC may be still relatively more immature at this age (Kostović et al. 2019; Kostović et al. 2021). The difference in network effects between sleep states needs to be sought from their assumed developmental functions. Although little is directly shown in the human infants, the recent animal experiments suggest that AS is crucial for activity-dependent network organization of, e.g., body maps (Blumberg et al. 2020b; Del Rio-Bermudez et al. 2020). Prior animal work has also shown that long-range PPC connectivity in AS is frequency-specific (Del Rio-Bermudez and Blumberg 2018; Del Rio-Bermudez et al. 2020). During QS, instead, brain is occupied by training the precise short-range connectivity (Brockmann et al. 2011; An et al. 2014). Future experimental studies are needed to examine the mechanistic underpinnings of the frequency ranges indicated in our present work.

The state-dependent reorganization of functional connectivity reflects brain network flexibility (Zalesky et al. 2014; Tokariev et al. 2019a), which enables diverse functions (Zalesky et al. 2014; Bassett et al. 2015; Hearne et al. 2017; Stevner et al. 2019). We observed that in utero exposure to AED results in a marked change in how cortical states reconfigure between AS and QS. Previous research has suggested that in utero exposure to AED has long-lasting neurobehavioral effects (Meador et al. 2009; Meador et al. 2013; Inoyama and Meador 2015; Videman et al. 2016a; Kellogg and Meador 2017). Our work identifies a specific mediator, namely a link between frontally connected functional networks in the delta–theta frequency range and neurodevelopment (Fig. 3C). In line with previous work (Tokariev et al. 2019a), the current results suggest that the magnitude of network change (i.e., the amount of functional discrimination between sleep states) may link to clinical performance. Together, our results suggest that the directions of brain-behavior correlations are not only frequency-specific, but they may link to the developmental roles of the sleep states: The AS supports motor-sensory development (Del Rio-Bermudez and Blumberg 2018; Sokoloff et al. 2021), whereas QS is thought it consolidate higher order cognitive functions (Graven and Browne 2008; Friedrich et al. 2020). Thus, the networks with stronger connectivity in AS related positively correlated to motor performance, whereas the networks with stronger connectivity in QS are negatively correlated to cognitive performance. These considerations support the idea that early development of motor and cognitive functions is supported by distinct subnetworks in the infant brain.

The present results show associations between network effects and compounded neurodevelopmental test scores at term age and at 2 years of age. These scales are used in individual clinical assessment, as well as in clinical research (Del Rosario et al. 2021). The scores of these scales provide relevant early outcomes within the limits of neurobehavioral performance available at the given ages. However, these scores cannot be used to predict detailed, long-term neurocognitive development (Bode et al. 2014; Månsson et al. 2019). Future prospective studies with longer term follow-up times are needed to establish the predictive value of early changes in networks activity on brain functions.

Future preclinical work could build on the present findings by assessing the neural mechanisms underpinning the observed effects. A candidate mechanism leading to the observed macroscopic network effects is the modulation of fetal spontaneous neural activity (Luhmann et al. 2016; Molnár et al. 2020; Hanganu-Opatz et al. 2021). AED administered to the mother crosses the placenta and the blood–brain barrier (Videman et al. 2016b), which may result in reducing fetal neuronal activity and large-scale synchrony via changes in ion channels’ function (Macdonald and Kelly 1995; Mantegazza et al. 2010; Åberg et al. 2013; McCorry and Bromley 2015) and/or neurotransmitter release (De Smedt et al. 2007; Shaw et al. 2020). Accordingly, the modulation of fetal neuronal activity by pharmacological (Åberg et al. 2013; Creeley 2016; Lotfullina and Khazipov 2018; Benninger et al. 2020; Chini et al. 2020; Mizuno et al. 2021), or pathological (Kirischuk et al. 2017; Cheyne et al. 2019) means has been linked to neuronal cell death and a host of physiological changes during early brain development (Åberg et al. 2013; Brackenbury et al. 2013; Granato and Dering 2018; Chini et al. 2020; Shaw et al. 2020). Therefore, experimental evidence supports two possible roles for developmental AED effects: a direct toxic effect of AED on cortical neurons or a secondary effect arising from an attenuated neuronal activity during in utero development. Our past (Videman et al. 2016b) and current results favors the latter mechanism. However, prospective animal research with comparable EEG metrics is needed to fully disclose cellular mechanisms.

The observed drug-specific effects on cortical networks supporting AS and QS cannot be directly informed by adult AED-related pharmacodynamic data (De Smedt et al. 2007). However, recent preclinical studies have suggested substantial changes in molecular and cellular mechanisms following the exposure to distinct AEDs. For example, alterations in the ion channels’ expression and neurotransmitter systems’ activity have been detected throughout rodents’ neurodevelopment (Klingler et al. 2021). Interestingly, this developmentally changing neuroanatomical and physiological landscape supports the emergence of distinct disease phenotypes, including developmental channelopathies (Smith and Walsh 2020). Accordingly, differences in sleep-related connectivity patterns between AEDs are likely caused by the complex time-varying interplay between neurophysiological mechanisms supporting fetal neurodevelopmental and maternal drug treatment during pregnancy.

Our mapping of infant’s cytoarchitectonic profiles (Conel 1941) suggests a link between cellular mechanisms and AED-specific changes in cortical networks activity as a function of AS and QS. These findings add a neonatal perspective to our fledgling understanding of the relationship between genetic, molecular, and cellular brain architectures and measures of neural activity, and functional connectivity (Zhang et al. 2017; Demirtas et al. 2019; Fulcher et al. 2019; Gomez et al. 2019; Paquola et al. 2019; Shine et al. 2019; Paquola et al. 2020; Beul et al. 2021). In the adult human brain, the distribution of cell types across the neocortex aligns with cortical microcircuitry and large-scale cortical networks activity measured by invasive electrical recording and functional MRI (Paquola et al. 2020). Physiological challenges, such as prematurity (Van der Veeken et al. 2020), maternal anesthesia (Van der Veeken et al. 2019), and preconception stress (Jenkins et al. 2018) can cause a reduction in neuronal density. Our findings suggest a targeted effect of AED on the neural substrate supporting the emergence of state-specific cortical networks. In addition to the association with infant cerebral cytoarchitecture (Shankle et al. 2000; Kostović et al. 2019), the topology of the “LEV” and “OXC” networks overlap with the cellular architecture of neural circuits in the adult brain (Paquola et al. 2019), including the principal axes of neurotransmitter receptor densities (Goulas et al. 2021). Further work characterizing the fetal structural and functional organizational principles of the brain (Hanganu-Opatz et al. 2021; Klingler et al. 2021) is needed to better understand the nature of the observed AED-specific effects. This work also provides a framework for detecting adverse effects of other psychotropic medications commonly used during pregnancy, as well as identifying fundamental mechanisms of the healthy development of functional brain networks in the newborn.

## Supplementary Material

Supplementary material can be found at *Cerebral Cortex* online.

## Funding

Academy of Finland (Suomen Akatemia): 321235, 313242, 288220, 310445; Sigrid Jusélius Foundation (Sigrid Juséliuksen Säätiö); Foundation for Pediatric Research (Lastentautien Tutkimussäätiö); and Finnish Brain Foundation (Suomen Aivosäätiö). L.C. was supported by National Health and Medical Research Council (1138711 and 2001283). M.P.v.d.H. was supported by an ALW open (ALWOP.179) and VIDI (452-16-015) grant from the Netherlands Organization for Scientific Research (NWO) and an ERC Consolidator of the European Research Council (101001062).

## Notes

Authors would like to thank Dr William R. Shankle for sharing Conel histological data collected and organized into Cyberchild database. We also thank Dr Miloš Judaš and Dr Goran Sedmak for providing Nissl staining of infant cortex. *Conflict of Interest*: None declared.

## Supplementary Material

Supplementary_material_AED_Tokariev_bhab338

## References

[ref1] Åberg E, Holst S, Neagu A, Ögren SO, Lavebratt C. 2013. Prenatal exposure to carbamazepine reduces hippocampal and cortical neuronal cell population in new-born and young mice without detectable effects on learning and memory. PLoS One. 8:e80497. 24244693 10.1371/journal.pone.0080497 PMC3828387

[ref2] An S, Kilb W, Luhmann HJ. 2014. Sensory-evoked and spontaneous gamma and spindle bursts in neonatal rat motor cortex. J Neurosci. 34:10870–10883. 25122889 10.1523/JNEUROSCI.4539-13.2014 PMC6705262

[ref3] André M, Lamblin MD, d'Allest AM, Curzi-Dascalova L, Moussalli-Salefranque F, S Nguyen The T, Vecchierini-Blineau MF, Wallois F, Walls-Esquivel E, Plouin P. 2010. Electroencephalography in premature and full-term infants. Developmental features and glossary. Neurophysiol Clin. 40:59–124. 20510792 10.1016/j.neucli.2010.02.002

[ref4] Bassett DS, Yang M, Wymbs NF, Grafton ST. 2015. Learning-induced autonomy of sensorimotor systems. Nat Neurosci. 18:744–751. 25849989 10.1038/nn.3993 PMC6368853

[ref5] Bayley N . 2006. Bayley Scales of Infant and Toddler Development. San Antonio, TX: Harcourt Assessment.

[ref6] Benders MJ, Palmu K, Menache C, Borradori-Tolsa C, Lazeyras F, Sizonenko S, Dubois J, Vanhatalo S, Hüppi PS. 2015. Early brain activity relates to subsequent brain growth in premature infants. Cereb Cortex. 25:3014–3024. 24867393 10.1093/cercor/bhu097

[ref7] Benninger KL, Borghese T, Kovalcik JB, Moore-Clingenpeel M, Isler C, Bonachea EM, Stark AR, Patrick SW, Maitre NL. 2020. Prenatal exposures are associated with worse neurodevelopmental outcomes in infants with neonatal opioid withdrawal syndrome. Front Pediatr. 8:462. 32974241 10.3389/fped.2020.00462 PMC7481438

[ref8] Beul SF, Goulas A, Hilgetag CC. 2021. An architectonic type principle in the development of laminar patterns of cortico-cortical connections. Brain Struct Funct. 226:979–987. 33559742 10.1007/s00429-021-02219-6 PMC8036174

[ref9] Bikbaev A, Ciuraszkiewicz-Wojciech A, Heck J, Klatt O, Freund R, Mitlohner J, Enrile Lacalle S, Sun M, Repetto D, Frischknecht R et al. 2020. Auxiliary alpha2delta1 and alpha2delta3 subunits of calcium channels drive excitatory and inhibitory neuronal network development. J Neurosci. 40:4824–4841. 32414783 10.1523/JNEUROSCI.1707-19.2020 PMC7326358

[ref10] Blumberg MS, Dooley JC, Sokoloff G. 2020a. The developing brain revealed during sleep. Curr Opin Physio. 15:14–22. 10.1016/j.cophys.2019.11.002 PMC7450535 32864534

[ref11] Blumberg MS, Lesku JA, Libourel PA, Schmidt MH, Rattenborg NC. 2020b. What is REM sleep? Curr Biol. 30:R38–r49. 31910377 10.1016/j.cub.2019.11.045 PMC6986372

[ref12] Bode MM, D'Eugenio DB, Mettelman BB, Gross SJ. 2014. Predictive validity of the Bayley, third edition at 2 years for intelligence quotient at 4 years in preterm infants. J Dev Behav Pediatr. 35:570–575. 25370298 10.1097/DBP.0000000000000110

[ref13] Brackenbury WJ, Yuan Y, O'Malley HA, Parent JM, Isom LL. 2013. Abnormal neuronal patterning occurs during early postnatal brain development of Scn1b-null mice and precedes hyperexcitability. Proc Natl Acad Sci USA. 110:1089–1094. 23277545 10.1073/pnas.1208767110 PMC3549092

[ref14] Brockmann MD, Poschel B, Cichon N, Hanganu-Opatz IL. 2011. Coupled oscillations mediate directed interactions between prefrontal cortex and hippocampus of the neonatal rat. Neuron. 71:332–347. 21791291 10.1016/j.neuron.2011.05.041

[ref15] Cheyne JE, Zabouri N, Baddeley D, Lohmann C. 2019. Spontaneous activity patterns are altered in the developing visual cortex of the Fmr1 knockout mouse. Front Neural Circuits. 13:57. 31616256 10.3389/fncir.2019.00057 PMC6775252

[ref16] Chini M, Popplau JA, Lindemann C, Carol-Perdiguer L, Hnida M, Oberlander V, Xu X, Ahlbeck J, Bitzenhofer SH, Mulert C et al. 2020. Resolving and rescuing developmental Miswiring in a mouse model of cognitive impairment. Neuron. 105(60–74):e67. 10.1016/j.neuron.2019.09.042 PMC6953432 31733940

[ref17] Conel JLR . 1941. The postnatal development of the human cerebral cortex: The cortex of the one-month infant. Oxford, England: Harvard Univ. Press.

[ref18] Creeley CE . 2016. From drug-induced developmental neuroapoptosis to pediatric anesthetic neurotoxicity-where are we now? Brain Sci. 6:32. 10.3390/brainsci6030032 PMC5039461 27537919

[ref19] Dale AM, Liu AK, Fischl BR, Buckner RL, Belliveau JW, Lewine JD, Halgren E. 2000. Dynamic statistical parametric mapping: combining fMRI and MEG for high-resolution imaging of cortical activity. Neuron. 26:55–67. 10798392 10.1016/s0896-6273(00)81138-1

[ref20] De Smedt T, Raedt R, Vonck K, Boon P. 2007. Levetiracetam: the profile of a novel anticonvulsant drug-part I: preclinical data. CNS Drug Rev. 13:43–56. 17461889 10.1111/j.1527-3458.2007.00004.x PMC6494143

[ref21] Del Rio-Bermudez C, Blumberg MS. 2018. Active sleep promotes functional connectivity in developing sensorimotor networks. Bioessays. 40:e1700234–e1700234. 29508913 10.1002/bies.201700234 PMC6247910

[ref22] Del Rio-Bermudez C, Kim J, Sokoloff G, Blumberg MS. 2020. Active sleep promotes coherent oscillatory activity in the cortico-hippocampal system of infant rats. Cereb Cortex. 30:2070–2082. 31922194 10.1093/cercor/bhz223 PMC7175014

[ref23] Del Rosario C, Slevin M, Molloy EJ, Quigley J, Nixon E. 2021. How to use the Bayley scales of infant and toddler development. Arch Dis Child Educ Pract Ed. 106:108–112. 32859738 10.1136/archdischild-2020-319063

[ref24] Demirtas M, Burt JB, Helmer M, Ji JL, Adkinson BD, Glasser MF, Van Essen DC, Sotiropoulos SN, Anticevic A, Murray JD. 2019. Hierarchical heterogeneity across human cortex shapes large-scale neural dynamics. Neuron. 101:1181, e1113–1194. 30744986 10.1016/j.neuron.2019.01.017 PMC6447428

[ref25] Despotovic I, Cherian PJ, De Vos M, Hallez H, Deburchgraeve W, Govaert P, Lequin M, Visser GH, Swarte RM, Vansteenkiste E et al. 2013. Relationship of EEG sources of neonatal seizures to acute perinatal brain lesions seen on MRI: a pilot study. Hum Brain Mapp. 34:2402–2417. 22522744 10.1002/hbm.22076 PMC6870156

[ref26] Destexhe A, Sejnowski TJ. 2003. Interactions between membrane conductances underlying thalamocortical slow-wave oscillations. Physiol Rev. 83:1401–1453. 14506309 10.1152/physrev.00012.2003 PMC2927823

[ref27] Dewell RB, Gabbiani F. 2019. Active membrane conductances and morphology of a collision detection neuron broaden its impedance profile and improve discrimination of input synchrony. J Neurophysiol. 122:691–706. 31268830 10.1152/jn.00048.2019 PMC6734408

[ref28] Dooley JC, Sokoloff G, Blumberg MS. 2019. Behavioral states modulate sensory processing in early development. Curr Sleep Med Rep. 5:112–117. 31662954 10.1007/s40675-019-00144-z PMC6818957

[ref29] Dubowitz LMS, Dubowitz V, Mercuri E. 1999. The neurological assessment of the pre-term & full-term newborn infant. London: Mac Keith Press.

[ref30] Engel AK, Gerloff C, Hilgetag CC, Nolte G. 2013. Intrinsic coupling modes: multiscale interactions in ongoing brain activity. Neuron. 80:867–886. 24267648 10.1016/j.neuron.2013.09.038

[ref31] Friedrich M, Mölle M, Friederici AD, Born J. 2020. Sleep-dependent memory consolidation in infants protects new episodic memories from existing semantic memories. Nat Commun. 11:1298. 32157080 10.1038/s41467-020-14850-8 PMC7064567

[ref32] Fulcher BD, Murray JD, Zerbi V, Wang XJ. 2019. Multimodal gradients across mouse cortex. Proc Natl Acad Sci U S A. 116:4689–4695. 30782826 10.1073/pnas.1814144116 PMC6410879

[ref33] Gomez J, Zhen Z, Weiner KS. 2019. Human visual cortex is organized along two genetically opposed hierarchical gradients with unique developmental and evolutionary origins. PLoS Biol. 17:e3000362. 31269028 10.1371/journal.pbio.3000362 PMC6634416

[ref34] Goulas A, Changeux JP, Wagstyl K, Amunts K, Palomero-Gallagher N, Hilgetag CC. 2021. The natural axis of transmitter receptor distribution in the human cerebral cortex. Proc Natl Acad Sci U S A. 118:e2020574118. 33452137 10.1073/pnas.2020574118 PMC7826352

[ref35] Gramfort A, Papadopoulo T, Olivi E, Clerc M. 2010. OpenMEEG: opensource software for quasistatic bioelectromagnetics. Biomed Eng Online. 9:45. 20819204 10.1186/1475-925X-9-45 PMC2949879

[ref36] Granato A, Dering B. 2018. Alcohol and the developing brain: why neurons die and how survivors change. Int J Mol Sci. 19:2992. 10.3390/ijms19102992 PMC6213645 30274375

[ref37] Graven SN, Browne JV. 2008. Sleep and brain development: the critical role of sleep in fetal and early neonatal brain development. Newborn Infant Nurs Rev. 8:173–179.

[ref38] Hanganu-Opatz IL, Butt SJB, Hippenmeyer S, De Marco Garcia NV, Cardin JA, Voytek B, Muotri AR. 2021. The logic of developing neocortical circuits in health and disease. J Neurosci. 41:813–822. 33431633 10.1523/JNEUROSCI.1655-20.2020 PMC7880298

[ref39] Hearne LJ, Cocchi L, Zalesky A, Mattingley JB. 2017. Reconfiguration of brain network architectures between resting-state and complexity-dependent cognitive reasoning. J Neurosci. 37:8399–8411. 28760864 10.1523/JNEUROSCI.0485-17.2017 PMC6596866

[ref40] Inoyama K, Meador KJ. 2015. Cognitive outcomes of prenatal antiepileptic drug exposure. Epilepsy Res. 114:89–97. 26088891 10.1016/j.eplepsyres.2015.04.016 PMC4475275

[ref41] Jenkins S, Harker A, Gibb R. 2018. Maternal preconception stress alters prefrontal cortex development in long-Evans rat pups without changing maternal care. Neuroscience. 394:98–108. 30366025 10.1016/j.neuroscience.2018.10.023

[ref42] Judaš M, Šimić G, Petanjek Z, Jovanov-Milošević N, Pletikos M, Vasung L, Vukšić M, Kostović I. 2011. The Zagreb collection of human brains: a unique, versatile, but underexploited resource for the neuroscience community. Ann N Y Acad Sci. 1225(Suppl 1):E105–E130. 21599691 10.1111/j.1749-6632.2011.05993.x

[ref43] Kabdebon C, Leroy F, Simmonet H, Perrot M, Dubois J, Dehaene-Lambertz G. 2014. Anatomical correlations of the international 10-20 sensor placement system in infants. Neuroimage. 99:342–356. 24862070 10.1016/j.neuroimage.2014.05.046

[ref44] Kellogg M, Meador KJ. 2017. Neurodevelopmental effects of antiepileptic drugs. Neurochem Res. 42:2065–2070. 28424947 10.1007/s11064-017-2262-4 PMC6390972

[ref45] Kirischuk S, Sinning A, Blanquie O, Yang JW, Luhmann HJ, Kilb W. 2017. Modulation of neocortical development by early neuronal activity: physiology and pathophysiology. Front Cell Neurosci. 11:379. 29238291 10.3389/fncel.2017.00379 PMC5712676

[ref46] Klingler E, Francis F, Jabaudon D, Cappello S. 2021. Mapping the molecular and cellular complexity of cortical malformations. Science. 371:eaba4517. 33479124 10.1126/science.aba4517

[ref47] Koolen N, Dereymaeker A, Rasanen O, Jansen K, Vervisch J, Matic V, Naulaers G, De Vos M, Van Huffel S, Vanhatalo S. 2016. Early development of synchrony in cortical activations in the human. Neuroscience. 322:298–307. 26876605 10.1016/j.neuroscience.2016.02.017 PMC4819727

[ref48] Kostović I, Radoš M, Kostović-Srzentić M, Krsnik Ž. 2021. Fundamentals of the development of connectivity in the human fetal brain in late gestation: from 24 weeks gestational age to term. J Neuropathol Exp Neurol. 80:393–414. 33823016 10.1093/jnen/nlab024 PMC8054138

[ref49] Kostović I, Sedmak G, Judaš M. 2019. Neural histology and neurogenesis of the human fetal and infant brain. Neuroimage. 188:743–773. 30594683 10.1016/j.neuroimage.2018.12.043

[ref50] Lotfullina N, Khazipov R. 2018. Ethanol and the developing brain: inhibition of neuronal activity and Neuroapoptosis. Neuroscientist. 24:130–141. 28580823 10.1177/1073858417712667

[ref51] Luhmann HJ, Sinning A, Yang JW, Reyes-Puerta V, Stüttgen MC, Kirischuk S, Kilb W. 2016. Spontaneous neuronal activity in developing neocortical networks: from single cells to large-scale interactions. Front Neural Circuits. 10:40. 27252626 10.3389/fncir.2016.00040 PMC4877528

[ref52] Macdonald RL, Kelly KM. 1995. Antiepileptic drug mechanisms of action. Epilepsia. 36(Suppl 2):S2–S12. 10.1111/j.1528-1157.1995.tb05996.x 8784210

[ref53] Månsson J, Stjernqvist K, Serenius F, Ådén U, Källén K. 2019. Agreement between Bayley-III measurements and WISC-IV measurements in typically developing children. J Psychoeduc Assess. 37:603–616.

[ref54] Mantegazza M, Curia G, Biagini G, Ragsdale DS, Avoli M. 2010. Voltage-gated sodium channels as therapeutic targets in epilepsy and other neurological disorders. Lancet Neurol. 9:413–424. 20298965 10.1016/S1474-4422(10)70059-4

[ref55] McCorry D, Bromley R. 2015. Does in utero exposure of antiepileptic drugs lead to failure to reach full cognitive potential? Seizure. 28:51–56. 25819874 10.1016/j.seizure.2015.01.019

[ref56] Meador KJ, Baker GA, Browning N, Clayton-Smith J, Combs-Cantrell DT, Cohen M, Kalayjian LA, Kanner A, Liporace JD, Pennell PB et al. 2009. Cognitive function at 3 years of age after fetal exposure to antiepileptic drugs. N Engl J Med. 360:1597–1605. 19369666 10.1056/NEJMoa0803531 PMC2737185

[ref57] Meador KJ, Baker GA, Browning N, Cohen MJ, Bromley RL, Clayton-Smith J, Kalayjian LA, Kanner A, Liporace JD, Pennell PB et al. 2013. Fetal antiepileptic drug exposure and cognitive outcomes at age 6 years (NEAD study): a prospective observational study. Lancet Neurol. 12:244–252. 23352199 10.1016/S1474-4422(12)70323-X PMC3684942

[ref58] Mizuno H, Rao MS, Mizuno H, Sato T, Nakazawa S, Iwasato T. 2021. NMDA receptor enhances correlation of spontaneous activity in neonatal barrel cortex. J Neurosci. 41:1207–1217. 33372060 10.1523/JNEUROSCI.0527-20.2020 PMC7888224

[ref59] Molnár Z, Luhmann HJ, Kanold PO. 2020. Transient cortical circuits match spontaneous and sensory-driven activity during development. Science. 370. 10.1126/science.abb2153 PMC8050953 33060328

[ref60] Odabaee M, Tokariev A, Layeghy S, Mesbah M, Colditz PB, Ramon C, Vanhatalo S. 2014. Neonatal EEG at scalp is focal and implies high skull conductivity in realistic neonatal head models. Neuroimage. 96:73–80. 24736169 10.1016/j.neuroimage.2014.04.007

[ref61] Palva JM, Wang SH, Palva S, Zhigalov A, Monto S, Brookes MJ, Schoffelen JM, Jerbi K. 2018. Ghost interactions in MEG/EEG source space: a note of caution on inter-areal coupling measures. Neuroimage. 173:632–643. 29477441 10.1016/j.neuroimage.2018.02.032

[ref62] Paquola C, Seidlitz J, Benkarim O, Royer J, Klimes P, Bethlehem RAI, Lariviere S, Vos de Wael R, Rodriguez-Cruces R, Hall JA et al. 2020. A multi-scale cortical wiring space links cellular architecture and functional dynamics in the human brain. PLoS Biol. 18:e3000979. 33253185 10.1371/journal.pbio.3000979 PMC7728398

[ref63] Paquola C, Vos De Wael R, Wagstyl K, Bethlehem RAI, Hong SJ, Seidlitz J, Bullmore ET, Evans AC, Misic B, Margulies DS et al. 2019. Microstructural and functional gradients are increasingly dissociated in transmodal cortices. PLoS Biol. 17:e3000284. 31107870 10.1371/journal.pbio.3000284 PMC6544318

[ref64] Rama S, Zbili M, Bialowas A, Fronzaroli-Molinieres L, Ankri N, Carlier E, Marra V, Debanne D. 2015. Presynaptic hyperpolarization induces a fast analogue modulation of spike-evoked transmission mediated by axonal sodium channels. Nat Commun. 6:10163. 26657943 10.1038/ncomms10163 PMC4682119

[ref65] Scholtens LH, de Reus MA, de Lange SC, Schmidt R, van den Heuvel MP. 2018. An MRI Von Economo - Koskinas atlas. Neuroimage. 170:249–256. 28040542 10.1016/j.neuroimage.2016.12.069

[ref66] Shankle WR, Landing BH, Rafii MS, Hara J, Fallon JH, Romney AK, Boyd JP. 2000. CYBERCHILD: a database of the microscopic development of the postnatal human cerebral cortex from birth to 72 months. Neurocomputing. 32:1109–1114.

[ref67] Shaw JC, Crombie GK, Zakar T, Palliser HK, Hirst JJ. 2020. Perinatal compromise contributes to programming of GABAergic and glutamatergic systems leading to long-term effects on offspring behaviour. J Neuroendocrinol. 32:e12814. 31758712 10.1111/jne.12814

[ref68] Shine JM, Breakspear M, Bell PT, Ehgoetz Martens KA, Shine R, Koyejo O, Sporns O, Poldrack RA. 2019. Human cognition involves the dynamic integration of neural activity and neuromodulatory systems. Nat Neurosci. 22:289–296. 30664771 10.1038/s41593-018-0312-0

[ref69] Siems M, Siegel M. 2020. Dissociated neuronal phase- and amplitude-coupling patterns in the human brain. Neuroimage. 209:116538. 31935522 10.1016/j.neuroimage.2020.116538 PMC7068703

[ref70] Smith RS, Walsh CA. 2020. Ion Channel functions in early brain development. Trends Neurosci. 43:103–114. 31959360 10.1016/j.tins.2019.12.004 PMC7092371

[ref71] Sokoloff G, Dooley JC, Glanz RM, Wen RY, Hickerson MM, Evans LG, Laughlin HM, Apfelbaum KS, Blumberg MS. 2021. Twitches emerge postnatally during quiet sleep in human infants and are synchronized with sleep spindles. Curr Biol. 31:3426–3432.e3424. 34139191 10.1016/j.cub.2021.05.038 PMC8355086

[ref72] Stevner ABA, Vidaurre D, Cabral J, Rapuano K, Nielsen SFV, Tagliazucchi E, Laufs H, Vuust P, Deco G, Woolrich MW et al. 2019. Discovery of key whole-brain transitions and dynamics during human wakefulness and non-REM sleep. Nat Commun. 10:1035. 30833560 10.1038/s41467-019-08934-3 PMC6399232

[ref73] Tadel F, Baillet S, Mosher JC, Pantazis D, Leahy RM. 2011. Brainstorm: a user-friendly application for MEG/EEG analysis. Comput Intell Neurosci. 2011:879716. 21584256 10.1155/2011/879716 PMC3090754

[ref74] Thomason ME . 2020. Development of brain networks in utero: relevance for common neural disorders. Biol Psychiatry. 88:40–50. 32305217 10.1016/j.biopsych.2020.02.007 PMC7808399

[ref75] Tokariev A, Roberts JA, Zalesky A, Zhao X, Vanhatalo S, Breakspear M, Cocchi L. 2019a. Large-scale brain modes reorganize between infant sleep states and carry prognostic information for preterms. Nat Commun. 10:2619. 31197175 10.1038/s41467-019-10467-8 PMC6565810

[ref76] Tokariev A, Stjerna S, Lano A, Metsäranta M, Palva JM, Vanhatalo S. 2019b. Preterm birth changes networks of newborn cortical activity. Cereb Cortex. 29:814–826. 30321291 10.1093/cercor/bhy012

[ref77] Tokariev A, Vanhatalo S, Palva JM. 2016a. Analysis of infant cortical synchrony is constrained by the number of recording electrodes and the recording montage. Clin Neurophysiol. 127:310–323. 26122070 10.1016/j.clinph.2015.04.291

[ref78] Tokariev A, Videman M, Palva JM, Vanhatalo S. 2016b. Functional brain connectivity develops rapidly around term age and changes between vigilance states in the human newborn. Cereb Cortex. 26:4540–4550. 26405053 10.1093/cercor/bhv219

[ref79] Van der Veeken L, Gronlund S, Gerdtsson E, Holmqvist B, Deprest J, Ley D, Bruschettini M. 2020. Long-term neurological effects of neonatal caffeine treatment in a rabbit model of preterm birth. Pediatr Res. 87:1011–1018. 31812154 10.1038/s41390-019-0718-8

[ref80] Van der Veeken L, Van der Merwe J, Devroe S, Inversetti A, Galgano A, Bleeser T, Meeusen R, Rex S, Deprest J. 2019. Maternal surgery during pregnancy has a transient adverse effect on the developing fetal rabbit brain. Am J Obstet Gynecol. 221:355 e351–355 e319. 31336075 10.1016/j.ajog.2019.07.029

[ref81] Veroniki AA, Rios P, Cogo E, Straus SE, Finkelstein Y, Kealey R, Reynen E, Soobiah C, Thavorn K, Hutton B et al. 2017. Comparative safety of antiepileptic drugs for neurological development in children exposed during pregnancy and breast feeding: a systematic review and network meta-analysis. BMJ Open. 7:e017248. 10.1136/bmjopen-2017-017248 PMC5642793 28729328

[ref82] Videman M, Stjerna S, Roivainen R, Nybo T, Vanhatalo S, Gaily E, Leppanen JM. 2016a. Evidence for spared attention to faces in 7-month-old infants after prenatal exposure to antiepileptic drugs. Epilepsy Behav. 64:62–68. 27732918 10.1016/j.yebeh.2016.09.023

[ref84] Videman M, Tokariev A, Stjerna S, Roivainen R, Gaily E, Vanhatalo S. 2016b. Effects of prenatal antiepileptic drug exposure on newborn brain activity. Epilepsia. 57:252–262. 26705760 10.1111/epi.13281

[ref83] Videman M, Tokariev A, Saikkonen H, Stjerna S, Heiskala H, Mantere O, Vanhatalo S. 2017. Newborn brain function is affected by fetal exposure to maternal serotonin reuptake inhibitors. Cereb Cortex. 27:3208–3216. 27269962 10.1093/cercor/bhw153

[ref85] Vinck M, Oostenveld R, van Wingerden M, Battaglia F, Pennartz CM. 2011. An improved index of phase-synchronization for electrophysiological data in the presence of volume-conduction, noise and sample-size bias. Neuroimage. 55:1548–1565. 21276857 10.1016/j.neuroimage.2011.01.055

[ref86] Xia M, Wang J, He Y. 2013. Brain net viewer: a network visualization tool for human brain connectomics. PLoS One. 8:e68910. 23861951 10.1371/journal.pone.0068910 PMC3701683

[ref87] Zalesky A, Fornito A, Bullmore ET. 2010. Network-based statistic: identifying differences in brain networks. Neuroimage. 53:1197–1207. 20600983 10.1016/j.neuroimage.2010.06.041

[ref88] Zalesky A, Fornito A, Cocchi L, Gollo LL, Breakspear M. 2014. Time-resolved resting-state brain networks. Proc Natl Acad Sci U S A. 111:10341–10346. 24982140 10.1073/pnas.1400181111 PMC4104861

[ref89] Zhang Y, Larcher KM, Misic B, Dagher A. 2017. Anatomical and functional organization of the human substantia nigra and its connections. Elife. 6:e26653. 28826495 10.7554/eLife.26653 PMC5606848

