# Supplementary material for: Impact of In Utero Exposure to Antiepileptic Drugs on Neonatal Brain Function
Source: Cereb Cortex. 2021 Sep 29;32(11):2385–97. doi: 10.1093/cercor/bhab338 (PMC9157298; doi:10.1093/cercor/bhab338)
Supplement: Supplementary_material_AED_Tokariev_bhab338 [file supplementary_material_aed_tokariev_bhab338.pdf]

Supplementary material

Impact of *in utero* exposure to antiepileptic drugs on neonatal brain function

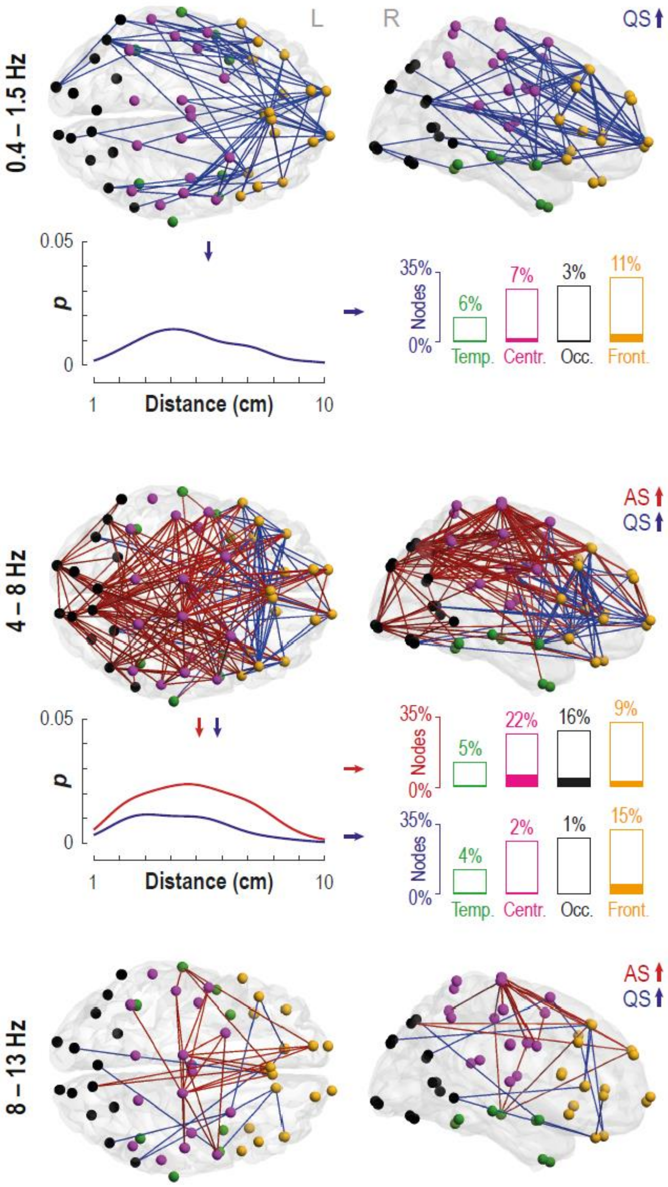

Supplementary Figure 1. Cortical networks linked to the main effect of sleep. *The main effect of sleep showed large-scale patterns of distinct increases in cortical connectivity in active sleep (AS, red) and quiet sleep (QS, blue). In the low delta (0.4–1.5 Hz), results highlight a frontally centred network encompassing long-range projections to parietal and occipital cortices at QS ($P_{FWE} < 0.0001$). The main effect of sleep in the theta regime (4–8 Hz) was characterised by two large-scale cortical networks: (i) a mid-posterior network in AS, and (ii) a frontal network supporting QS ($P_{FWE} < 0.0001$ for both networks). In the alpha frequency range (8–13 Hz), results highlight two sparse networks. The network associated with AS ($P_{FWE} = 0.004$) is anchored to central cortical regions. Conversely, the network linked to QS connect frontal to occipital cortices ($P_{FWE} = 0.048$). Probability (p) distribution curves reflect Euclidean distances between cortical areas. Bar plots show the proportion of nodes in four main cortical regions.*

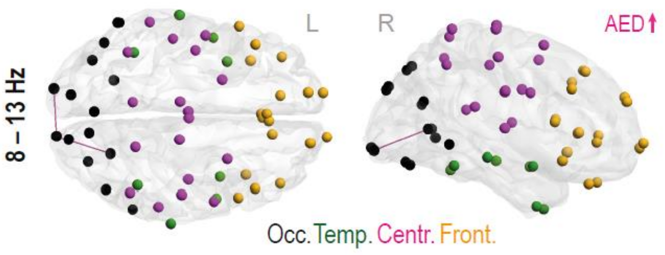

Supplementary Figure 2. Main effect of group in the alpha frequency band (8–13 Hz). *Connectivity in two posterior edges was stronger in AED than HC (P_{FWE} = 0.01; unpaired two-tailed t-test). The association of distinct cortical nodes to broader cortical regions is highlighted by different colours: occipital (black), temporal (green), central (purple), and frontal (orange).*

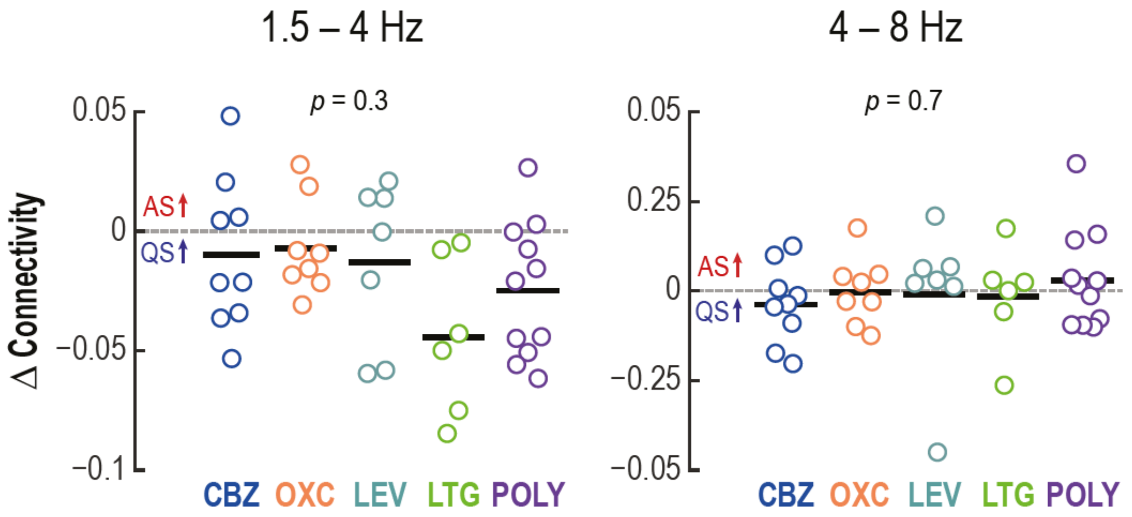

Supplementary Figure 3. AED-specific effects in the general interaction networks (Fig. 3A). *No significant differences in sleep-related changes in the global mean connectivity (Δ = AS − QS) were observed between different AED types (Kruskal-Wallis test). Each circle represents one infant. The AEDs are coded with the following colours: carbamazepine (CBZ, blue), oxcarbazepine (OXC, orange), levetiracetam (LEV, aquamarine), lamotrigine (LTG, green), and polytherapy (POLY, violet). Solid lines indicate the subgroup means.*

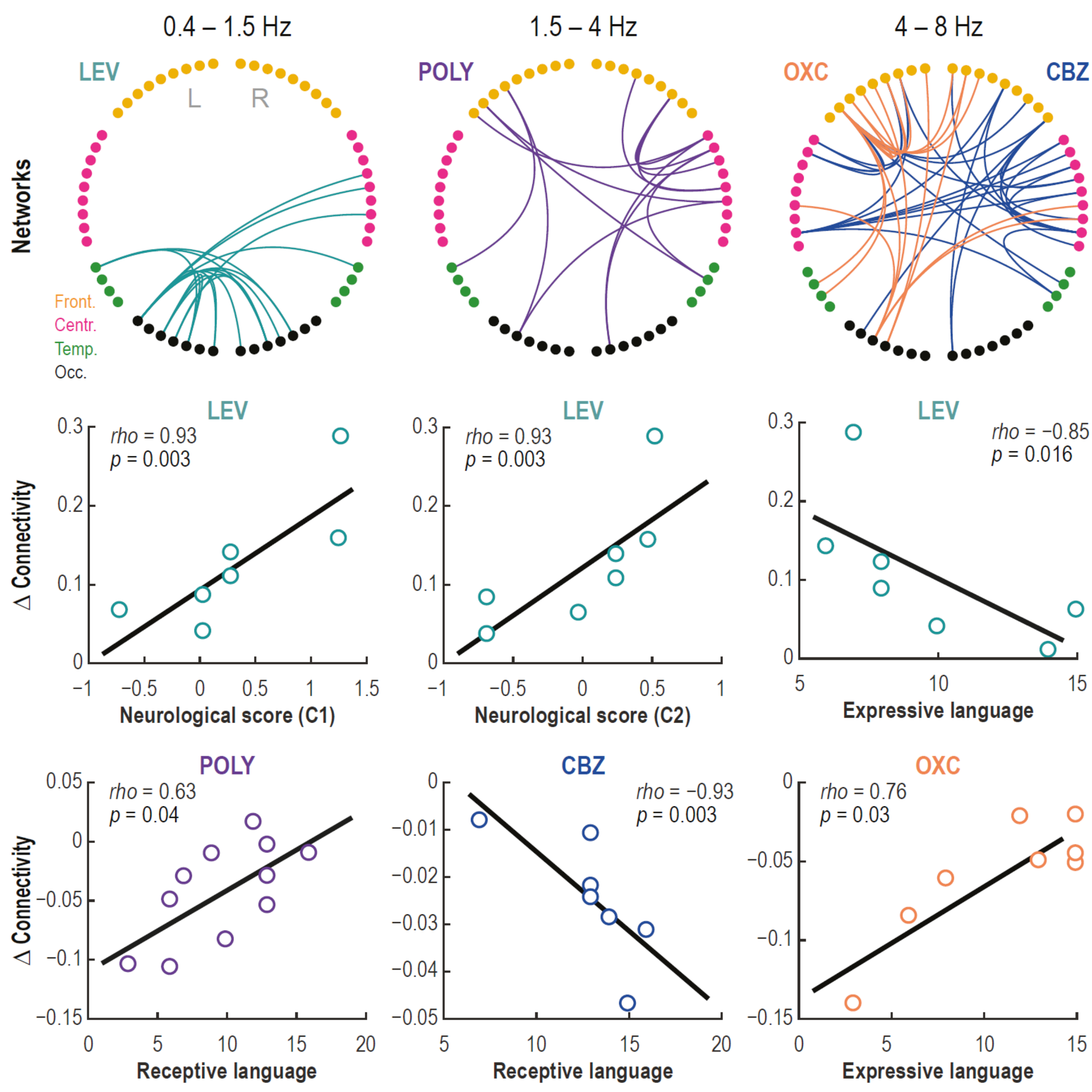

Supplementary Figure 4. Correlation between AED-related network dynamics as a function of sleep states and clinical outcomes. *The top graphs display frequency-resolved summaries of cortical drug-induced networks (sleep-by-group interactions; Fig. 4A) carrying prognostic information. The scatter plots show linear relationships between clinical outcome measures and the magnitude of network dynamics (i.e. AS minus QS) in given network. The network dynamics in the LEV subgroup were correlated to the compound neurological C1 and C2 scores at term age (for both tests rho = 0.93, p = 0.003), as well as to the expressive language performance at two years (rho = −0.85, p = 0.016). In the POLY and CBZ, changes in connectivity were associated with receptive language scores at two years (with rho = 0.63, p = 0.04 and rho = −0.93, p = 0.003 respectively). Finally, changes in sleep-related connectivity in the OXC subgroup correlated with expressive language scores at two years (rho = 0.76, p = 0.03). Linear correlations were estimated with two-tailed Spearman's correlation.*

Table 1: Network-to-outcome correlations

Score	1.5 – 4 Hz rho	1.5 – 4 Hz p	4 – 8 Hz rho	4 – 8 Hz p
**Newborn neurology (term age)**				
C1	−0.24	0.111	−0.13	0.373
C2	0.02	0.912	−0.36	0.015
**Neurocognitive development (2 years)**				
Cognitive	0.24	0.116	0.10	0.504
Receptive communication	0.25	0.101	0.17	0.274
Expressive communication	0.09	0.553	0.22	0.152
Fine motor	0.42	0.005	0.04	0.792

Supplementary Table 1 *Correlation tests (Spearman) between connectivity changes as a function of sleep (see Fig. 3) and neurological/neurocognitive scores for AED infants.*

Consideration of the EEG artefacts

EEG epochs containing major artefacts were excluded from the analysis. However, EEG recordings from sleeping newborn infants inevitably include some residual artefacts, such as muscle activity, head movements, respiration-related movements, and eye movements. In theory, they influence phase-phase correlation (PPC) measures. However, it is unlikely that they caused the observed findings due to several reasons: First, these artefacts are not likely to systematically differ between patient groups, hence the artefact effect should not appear in the group difference results. Second, respiration artefact causes spurious synchrony only at the respiration frequency (low delta) and mostly in the occipital and temporal areas that are more affected by the head movements. This is different from the frontally bound delta frequency findings in our work. Eye movements were previously shown to be very unlikely cause of artefacts in the PPC analysis(Tokariev et al. 2019). Muscle activity is intermittent and can be sometimes prominent in the frontal electrodes during active sleep, however these muscles do not possess the large-scale spatial structure and low frequency content to yield spurious PPC results (Freeman et al. 2003). Finally, movements are relatively rare and variable, and their frequency content is even lower than what is seen with respiration. Thus, it is unlikely that movements give rise to any PPC present in the EEG signal.

References

Freeman WJ, Holmes MD, Burke BC, Vanhatalo S. 2003. Spatial spectra of scalp EEG and EMG from awake humans. Clin Neurophysiol. 114:1053-1068.

Tokariev A, Stjerna S, Lano A, Metsäranta M, Palva JM, Vanhatalo S. 2019. Preterm Birth Changes Networks of Newborn Cortical Activity. Cereb Cortex. 29:814-826.
